# Exploring the Predictors of Rapid Eye Movement Sleep Behavior Disorder for Parkinson’s Disease Patients Using Classifier Ensemble

**DOI:** 10.3390/healthcare8020121

**Published:** 2020-05-01

**Authors:** Haewon Byeon

**Affiliations:** Department of Speech Language Pathology, School of Public Health, Honam University, 417, Eodeung-daero, Gwangsan-gu, Gwangju 62399, Korea; byeon@honam.ac.kr; Tel.: +82-10-7404-6969

**Keywords:** Parkinson’s disease dementia, cognitive function, rapid eye movement sleep behavior disorder, random forest, RBF artificial neural network, neuropsychological profile

## Abstract

The rapid eye movement sleep behavior disorder (RBD) of Parkinson’s disease (PD) patients can be improved with medications such as donepezil as long as it is diagnosed with a thorough medical examination, since identifying a high-risk group of RBD is a critical issue to treat PD. This study develops a model for predicting the high-risk groups of RBD using random forest (RF) and provides baseline information for selecting subjects for polysomnography. Subjects consisted of 350 PD patients (Parkinson’s disease with normal cognition (PD-NC) = 48; Parkinson’s disease with mild cognitive impairment (PD-MCI) = 199; Parkinson’s disease dementia (PDD) = 103) aged 60 years and older. This study compares the prediction performance of RF, discriminant analysis, classification and regression tree (CART), radial basis function (RBF) neural network, and logistic regression model to select a final model with the best model performance and presents the variable importance of the final model’s variable. As a result of analysis, the sensitivity of RF (79%) was superior to other models (discriminant analysis = 14%, CART = 32%, RBF neural network = 25%, and logistic regression = 51%). It was confirmed that age, the motor score of Untitled Parkinson’s Disease Rating (UPDRS), the total score of UPDRS, the age when a subject was diagnosed with PD first time, the Korean Mini Mental State Examination, and Korean Instrumental Activities of Daily Living, were major variables with high weight for predicting RBD. Among them, age was the most important factor. The model for predicting Parkinson’s disease RBD developed in this study will contribute to the screening of patients who should receive a video-polysomnography.

## 1. Introduction

Parkinson’s disease (PD) is defined as a neurodegenerative disease that shows distinctive motor symptoms such as bradykinesia, rigidity, tremor at rest, and postural instability. Although traditional PD diagnosis and treatment methods have been developed with focusing on these motor symptoms, recent studies tend to focus more on the non-motor symptoms of PD [1] because it has been reported that PD patients are highly likely to experience non-motor symptoms such as cognitive dysfunction, depression, and sleep behavior disorders [2].

The non-motor symptoms of PD can be divided into cognitive abnormalities, autonomic dysfunction, impaired olfaction, mood disorders (e.g., depression and anxiety), and sleep disorders [3]. Among them, sleep disorder is a representative non-motor symptom of Parkinson’s disease, and the rapid eye movement sleep behavior disorder (RBD) is frequently observed [4]. The International Classification of Sleep Disorders (3rd edition; 2014) [5] reported that RBD is characterized by excessive movements related to the content of the dream because the atonia of skeletal muscles, normally occurring during rapid eye movement (REM) sleep, is lost. Even though 0.5% of the population experience RBD [6], 15%–60% of PD patients suffer from RBD and frequently have sleep disorders [7,8]. Therefore, it is necessary to carefully observe the progress of Parkinson’s disease, even if RBD is not observed in the early stages.

Most dreams associated with RBD include negative emotions or aggressive content [9]. Consequently, patients are likely to scream or hurt their spouses (or dependents) while dreaming [10]. Therefore, it is necessary to diagnose and treat RBD as early as possible in order to protect PD patients and their spouses from trauma due to RBD. The diagnosis procedure of RBD is to first identify the symptoms of sleep behavior disorder by asking the spouse (or dependent) of a patient about the conditions of the patient and confirm RBD by conducting polysomnography for precise diagnosis [11]. However, since the symptoms of RBD frequently occur early in the morning [11], caregivers often do not find the symptom or often do not report the symptom to medical personnel because they take the symptom for granted. Moreover, it is limited to identifying subjects who need to receive a detailed test for RBD through interviewing because the number of senior citizens who live alone is increasing. The RBD of PD patients can be improved by administering benzodiazepine such as clonazepam [12] and donepezil [13]. However, taking a large amount over a long period of time may make the patients depend on the medication, and sudden decrease in dosage may cause withdrawal symptoms. Therefore, it is not recommended for neurologists to prescribe benzodiazepine for preventing PD patients from having RBD. Therefore, it is important to identify the high-risk group of RBD as soon as possible to treat RBD appropriately.

It has been recommended to develop a model to predict a high-risk group of RBD because it commonly occurs from the early stage of Parkinson’s disease [14] and is difficult to diagnose with the disease just by interviewing caregivers. However, the predictors of RBD are still not known well. Previous studies [12,15] suggested that medicinal factors (e.g., antidepressant, benzodiazepine family drugs, cholinesterase inhibitors, caffeine, and tramadol), disease factors (e.g., duration and severity of Parkinson’s disease), and age could be predictors. However, a study comparing Parkinson’s disease patients with healthy people is needed in order to discriminate the sleep behavior disorder of Parkinson’s disease using hospital registry data because little is known about neuropsychological characteristics of RBD of PD patients.

Until now, various fields including health science have used traditional statistical (classification) techniques (e.g., multiple logistic regression analysis and multiple discriminant analysis) and various algorithms (e.g., artificial neural network and decision tree) as machine learning techniques for predicting target variables [16]. However, these algorithms may have the following limitations depending on the characteristics of the dataset: (1) although regression analysis is effective for identifying individual risk factors, the selection of risk factors depends on researchers’ rule of thumb [17]; (2) artificial neural network and decision tree are vulnerable to overfitting, noise, and outliers [18]; and (3) the accuracy of decision trees is highly likely to vary depending on the type and quantity of input variables.

The ensemble technique has been used as a new technique more frequently instead of the conventional decision tree for developing a disease prediction model [19]. Among them, random forest (RF) is a classification algorithm proposed by Leo Breiman [18]. It is a representative ensemble-based machine learning technique that improves prediction accuracy and stability by using multiple decision trees. Even though it is known that the prediction performance of RF is higher than that of decision tree [20], prediction studies using biomarkers [21] and those using images [22] have been mainly conducted for evaluating diseases so far. Moreover, only a few RF-based studies utilize sociodemographic factors, questionnaire data such as health habits, or neuropsychological examination data [23]. This study develops a model for predicting the high-risk groups of Parkinson’s disease sleep behavior disorder using RF and provides baseline information for selecting subjects for polysomnography. In addition, the prediction performance of the developed RF model is compared with those of other supervised learning-based classifiers (e.g., classification and regression tree (CART), radial basis function (RBF) neural network, logistic regression analysis, and discriminant analysis) to verify the prediction efficiency of neuropsychological tests using disease data.

## 2. Methods and Materials

### 2.1. Subjects

The present study was carried out using the Parkinson’s Dementia Clinical Epidemiology Data (PADEM data) from the National Biobank of Korea, the Center for Disease Control and Prevention, the Republic of Korea (No. KBN-2019-005). This study was approved by the Research Ethics Review Board (No. KBN-2019-005), the National Biobank of Korea, and it was also approved by the Korea Centers for Disease Control and Prevention to use the data (No. KBN-2019-1327). The PADEM data were collected under the management of the Korea Centers for Disease Control and Prevention at 14 tertiary care organizations (university hospitals) from January to December in 2015. Health surveys were performed using computer-assisted personal interviews. The primary goal of the National Biobank of Korea is to advance biomedical research and public health (see [24] for further details of the National Biobank of Korea, including quality control programs). This study analyzed 350 PD patients (Parkinson’s disease with normal cognition (PD-NC) = 48; Parkinson’s disease with mild cognitive impairment (PD-MCI) = 199; Parkinson’s disease dementia (PDD) = 103) and all subjects were 60 years or older.

### 2.2. Measurement and Definition of Variables

The PADEM data were composed of sociodemographic factors (e.g., sex), environmental factors (e.g., exposure to pesticides), health behaviors (e.g., smoking habits), disease history (e.g., diabetes), exercise characteristics related to PD (e.g., rigidity), and neuropsychological profile (e.g., cognitive function). For RBD, sleep problems were measured by using basic clinical psychological evaluation including sleep and video-polysomnography. The measurements were diagnosed by a psychiatrist based on the criteria of the International Classification of Sleep Disorders (3rd edition; 2014) [5]. The input variables included age, gender, education, mainly used hand, family dementia history, family PD history, pack-years, coffee-drinking, mean coffee intake per day, coffee drinking period, pesticide exposure recognition, disease history, PD related motor signs, and neuropsychological profiles. The definitions of the variables are shown in Table 1.

### 2.3. Classification Algorithms

Machine learning algorithms are largely divided into supervised learning and unsupervised learning. Supervised learning is used when information on the outcome variables is known in advance and unsupervised learning is used when the target variables are not defined precisely or information on them is not available. Since this study knew the prevalence of the disease in advance through collecting patient information of a specific disease in advance, this study used supervised learning based classification algorithms such as RF, CART, RBF neural network model, logistic regression model, and discriminant analysis. All analyses were performed using R-version 3.5.1 (Foundation for Statistical Computing, Vienna, Austria).

**Figure 1:**
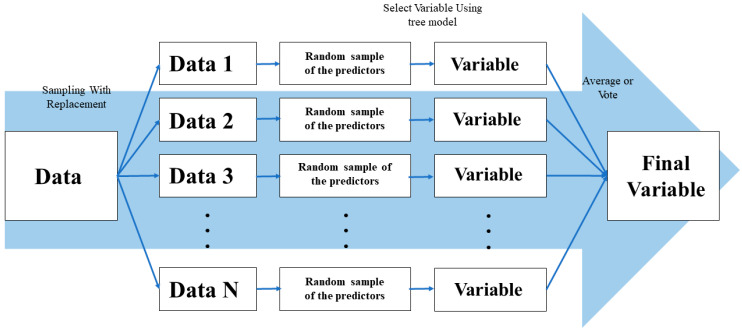
Concept of random forest.

**Figure 2:**
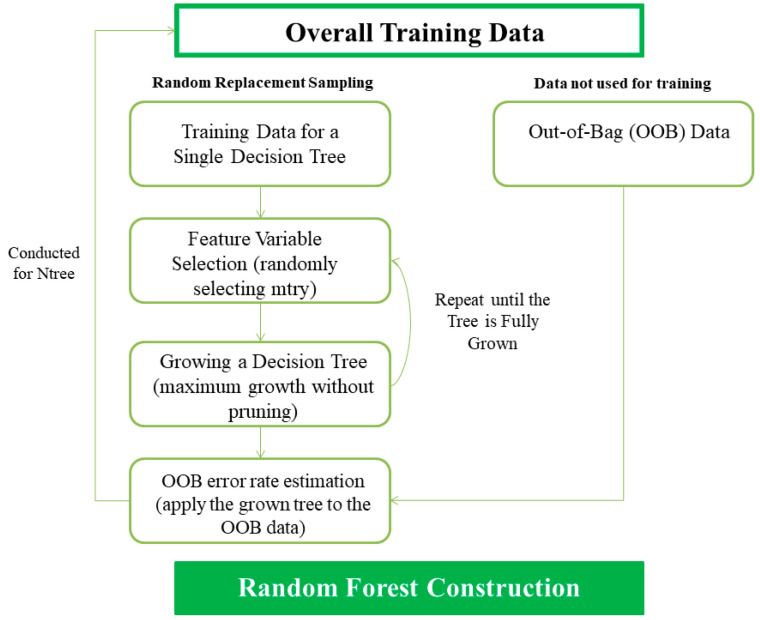
The procedure of developing a random forest (RF)-based prediction model.

RF is a meta-learning type machine learning technique of decision tree, which utilizes multiple decision trees instead of one decision tree. Each decision tree in RF is formed by randomly selected training data and input variables. In this case, the precision of each individual decision tree may be low, but the accuracy and stability of the RF increases as the predictions are combined [25]. Bootstrapping repeatedly generates samples equal to the size of the original sample through a replacement process. After multiple bootstrap samples are generated by applying this process, bagging creates each classification model for each bootstrap sample parallel. As a result, the prediction result of each model is calculated. Afterward, the prediction results of each model are combined using a voting method for classification and they are merged through averaging the numerical prediction [26]. RF is a technique designed to combine these prediction results after generating numerous decision trees by combining random input variables with the bagging (Figure 1). Since it has randomness for both the feature and instance, RF can be theoretically free from overfitting, is less affected by noise and outliers, and has high accuracy by lowering generalization errors. RF is more likely to have an elbow point that improves accuracy with an abrupt decrease of the slope when there are more trees. If an unimportant explanatory variable is selected, there is a higher probability that each tree becomes more complex. Therefore, this study set the number of mtry, the number of explanatory variable candidates in advance to minimize problems such as elbow points. The procedure of developing an RF-based prediction model is presented in Figure 2.

Step 1: Values for basic parameters to be applied to RF shall be determined. In the training stage, ntree, the total number of decision trees to construct RF must be determined in advance. In general, ntree is set to 500 or more to obtain the effect of a large sample size. It was set to 1000 in this study.

Step 2: *X_i_* (where, _I_ = number of bootstrap reiterations) shall be extracted for the bootstrap sample. *X_i_* shall be extracted as a random sample from the training data (*X*), and it shall be extracted by using sampling with the replacement method.

Step 3: The binary decision tree shall be grown using the bootstrap sample *X_i_*. The Gini impurity is used as a classification criterion for optimal partitioning. If an explanatory variable improves the classification performance of a model considerably, the Gini impurity of this variable will decrease, and the importance of this variable will increase. The function of the Gini impurity is presented in Equation (1).

(1) $$G\left(p_{1},\;p_{2},\;\cdots ,\;p_{J}\right)=\sum_{i=1}^{J}p_{i}\left(1-p_{i}\right)$$

Step 4: Prediction results shall be calculated. The error of out-of-bag (OOB) against the OOB data shall be calculated to confirm how well the predicted results match the actual results. OOB error is a method of measuring the prediction error of machine learning models utilizing bootstrap aggregating (bagging) to sub-sample data samples used for training.

Step 5: The procedures from Step 2 to Step 4 are repeated ntree times to finally construct an RF consisting of ntree decision trees. When the dependent variable is a continuous variable, the final predicted value of RF shall be a mean value. When it is a categorical variable, it shall be calculated by combining the predicted values of individual decision trees based on the results of the voting.

The prediction performance of the developed RF model was compared with those of other supervised learning-based classifiers such as CART, RBF neural network, discriminant analysis, and logistic regression analysis. CART involves a series of binary questions (yes or no) obtained from patient data [27]. These could entail a threshold for numeric parameters or the presence or absence of a specific state or combination of states. The algorithm iterates through the possible combinations to determine the optimal model. Neural networks are a set of algorithms, modeled loosely after the human brain, that are designed to recognize patterns [28]. Input values are multiplied by a weight and passed to the next layer of nodes. Each node has an activation function that derives an output-based input value passed to it by the previous node and weight. The weights are adjusted through successive iterations, or epochs, to achieve the optimal model. Discriminant analysis is frequently used to determine a linear combination of parameters that characterize or separate two or more classes of objects or events [29]. Logistic regression is a statistical model that in its basic form uses a logistic function to model a binary dependent variable. In regression analysis, logistic regression estimates the parameters of a logistic model, a form of binary regression [30].

### 2.4. Comparing the Accuracy of Sleep Behavior Disorder Prediction Model

For disease data defined as a binary type, there is a possibility of a class imbalance, indicating that the number of a specific class is more than that of another class [31]. The examined data had 47.7% of subjects with an RBD and 52.3% of subjects without RBD, showing similar ratios. Therefore, it was assumed that the analysis data of this study would not cause a classification asymmetry problem.

The prediction performance of the model was verified by considering the overall accuracy, sensitivity, and specificity together. Sensitivity is the prediction performance of RBD and specificity reflects the prediction performance of cases without RBD. Since the objective of this study was to develop a model to predict RBD, the overall accuracy and sensitivity were considered first to evaluate the prediction performance of the model. When two models had identical overall accuracy and sensitivity, specificity was considered to determine a better model. This study compared the prediction performance of RF, discriminant analysis, CART, RBF artificial neural network, and logistic regression model to select a final model with the best model performance and presented the variable importance of the final model’s variable. Moreover, a partial dependence plot was presented for an input variable with the highest variable importance to visually confirm the tendency of marginal effects on the response variable. The function of partial dependence is presented in Equation (2).

(2) $$f\left(x\right)=\frac{1}{n}\overset{n}{\underset{i=1}{\sum }}log\left(\frac{p_{1}\left(x,\;x_{ic}\right)}{p_{0}\left(x,x_{ic}\right)}\right)$$

In the above equation, *p*1 (*x, x_c_*) is Pr (*Y* = 1), calculated from a specific value of an interest variable (*x*) and fixed values of the remaining predictors (*x_c_*). This probability is calculated as the ratio classified as *Y* = 1 category in the corresponding random decision tree. In other words, partial dependence is the concept of log odds in the logit model and it is the mean after calculating the log odds from all observations (*i*).

## 3. Results

### 3.1. General Characteristics of Subjects

The general characteristics of the subjects are presented in Table 2. The mean age of the subjects was 68.6 years (SD = 8.9) and the mean age at the time of being diagnosed with Parkinson’s disease the first time was 68.0 years (SD = 8.6). Approximately 54.6% of the subjects were female, 61.7% of them were middle school graduates or below, 89.1% were smokers smoking more than 61 pack-years, and 59.0% did not have depression. Furthermore, 6.2% had a family history of Parkinson’s disease, 5.9% had a family history of Alzheimer’s disease, and 47.7% were diagnosed with RBD.

### 3.2. Results of Developing an RF-Based Parkinson’s Sleep Behavior Disorder Prediction Model

In the case of mtry, presenting the number of explanatory variables to be used in the decision tree constituting RF, this study applied all values from 5 to 15 and selected the value with the smallest OOB error rate. Table 3 shows the change in the OOB error rate according to the number of mtry (the number of explanatory variable candidates). This study selected 8 as the optimal number of mtry that generated the lowest OOB error rate (29.4%). When ntree was 1000 and mtry was 8, the prediction accuracy of RF was 71.5%.

### 3.3. Selection of the Final Prediction Model

Table 4 shows the results of the prediction performance of the verification data by the algorithm. The overall accuracy of the RBF artificial neural network was the highest (78%), but it showed the lowest sensitivity (25%) among the models predicting RBD. On the other hand, the overall accuracy of the RF was 71%, lower than that of the RBF artificial neural network, but the sensitivity of it was 79%, higher than that of the RBF artificial neural network (25%). As a result, RF was selected as the final prediction model with the best prediction among the RBD prediction models developed by this study.

### 3.4. Importance of Variables in the Final Model

**Figure 3:**
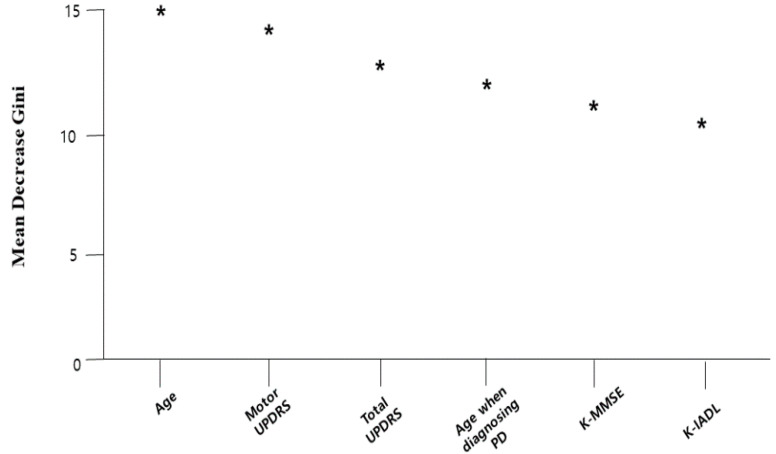
The normalized importance values in RF model. Mean decrease Gini means the degree of an explanatory variable’s effects on the classification accuracy of a model.

The normalized importance values in RF, the final model, are presented in Figure 3. It was confirmed that age, the motor score of Untitled Parkinson’s Disease Rating (UPDRS), the total score of UPDRS, the age when a subject was diagnosed with PD the first time, the Korean Mini Mental State Examination (K-MMSE) score, and the Korean Instrumental Activities of Daily Living (K-IADL) score were major variables with high weight for predicting RBD. Among them, age was the most important factor.

**Figure 4:**
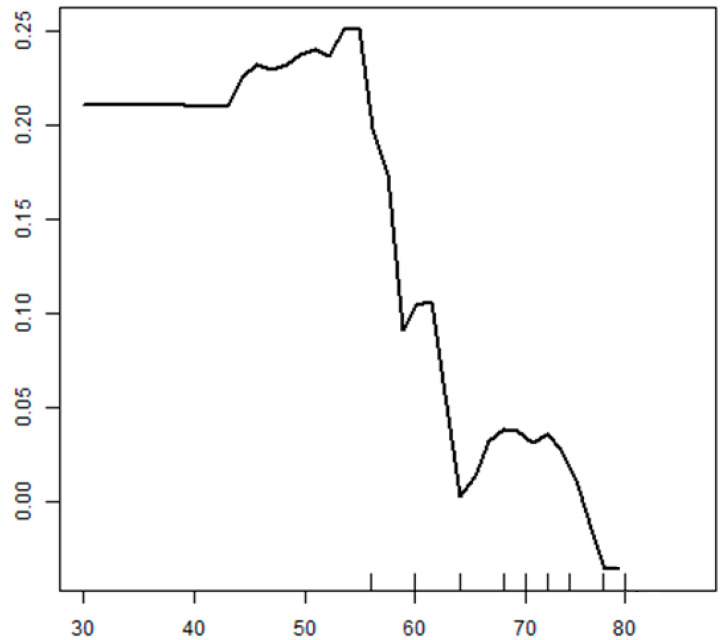
The partial dependence plot of age.

The partial dependence plot of “Age”, the factor with the highest variable importance in the Parkinson’s disease REM sleep prediction model, is presented in Figure 4. When the other factors (variables) were the same, the possibility of RBD increased as age became higher. In other words, it was confirmed that old age was an independently associated factor of Parkinson’s disease RBD.

## 4. Discussion

Early screening of RBD is important in health science because RBD is the most commonly occurring non-motor symptom in PD and it is highly likely to affect the quality of patients’ and caregivers’ lives. This study explored key discriminating indicators of RF-based RBD while considering sociodemographic factors, health habits, and neuropsychological tests and found that age, the motor score of UPDRS, the total score of UPDRS, the age when a subject was diagnosed with PD for the first time, K-MMSE score, and K-IADL score were the key predictors. Among them, age was the most important predictor. Lee et al. [32] reported that old age, a longer duration of PD, a more severe level of disability, and an increase of tremor score were significant risk predictors of RBD for PD patients using logistic regression analysis. Sixel-Döring et al. [33] also reported that older age, longer disease duration, and psychiatric comorbidity were significantly related with RBD.

RBD is frequently found in patients who suffer from Parkinson’s disease longer and have more progressed symptoms [15,34]. Moreover, it is known that the occurrence frequency of it is high in elderly patients [15,34]. However, the intensity of REM sleep behavior symptoms may not be distinctive for elderly patients because it tends to change as the underlying neurodegenerative change progresses [35]. In addition, the symptoms of RBD are identified by interviewing family members of Parkinson’s disease patients [11]. Moreover, through this process, it was determined whether to perform a detailed examination (polysomnography) or not [11]. If the symptoms are minor, it is difficult for the family to recognize them clearly. Therefore, it is limited to screen the subject of polysomnography by age alone. Based on the results of this study, it is possible to more accurately predict a high-risk group of RBD in elderly PD patients by conducting tests focusing on motor dysfunctions such as the motor score of UPDRS and total score of UPDRS, cognitive screening tests such as K-MMSE, and physical function tests such as K-IADL. However, additional epidemiological studies should be conducted to verify that aging and neuropsychological profiles are important predictors for distinguishing between AD and PDD. Although it has stronger prediction power than traditional regression models such as logistic regression, a disadvantage of machine learning is that it does not allow for interpretation of the derived results. Therefore, future studies should employ hybrid approaches combining random forests with other learning which have high predictive power and can interpret results.

Another finding of this study was that the prediction performance of RF was superior to that of CART or RBF artificial neural network as well as that of traditional statistical techniques such as discriminant analysis and regression analysis. The results of this study agreed with a previous study [36] that predicted the high-risk group of cognitive impairment in old age using RF. It is believed that the prediction performance of RF was better than that of traditional statistical techniques (e.g., regression analysis) or that of CART because RF was based on bootstrap aggregating (bagging), generating diverse trees from multiple bootstrap samples. In other words, while CART has a risk of overfitting because even an outlier is highly likely to be constructed as a node without exception, RF can prevent overfitting because it generates multiple bootstrap samples and has better prediction accuracy than decision tree [37]. RF showed high prediction performance even when the binomial classification was conducted using imbalanced data like disease data [36].

It is worthwhile to note that RF is also relatively free from the problems inherent (limitations) in basic classification algorithms such as artificial neural networks. For example, although an artificial neural network has high prediction accuracy, it is difficult to analyze a causal relationship, model learning is complex, and there is a possibility of overfitting [38]. On the other hand, RF is a very cost-effective algorithm that can learn quickly with little influence of outliers [39].

In particular, RF is suitable for analyzing big data due to the nature of the algorithm, which is an advantage. One of the most challenging tasks for applying machine learning techniques to a big data analysis environment is that it is necessary to divide the data and models for model construction for processing variance. RF is highly compatible with big data because the original algorithm of it is designed to properly divide data and input variables, learn multiple decision trees, and combine them later. Therefore, when analyzing multiple independent variables like disease big data or developing a model that predicts a target variable using data containing outliers, using RF will provide better accuracy and sensitivity than CART or artificial neural network.

The importance of this study was that it developed an RF model using sociodemographic characteristics and disease examination data such as a neuropsychological test and tested the prediction performance of machine learning techniques according to the classification algorithm. The limitations of the study are as follows. First, various approaches should be considered in the future to improve the performance of RF. The performance of RF can be related to the optimization of design parameters such as ntree or mtry. However, this study could not consider the parameter optimization of ntree. Future studies may be needed to conduct sensitivity analysis for the parameters of RF and optimize RF parameters. Secondly, when a large number of input variables are considered and/or a statistical model is applied in particular, it is necessary to deal with the multicollinearity problem more carefully. However, this study did not consider multicollinearity. Therefore, future studies should consider developing a predictive model while taking multicollinearity into account. Thirdly, this study did not consider how to deal with diversity and accuracy-based classifier selection and data imbalance problems. In particular, since this study did not observe the data imbalance of a target variable, it was impossible to identify the performance of machine learning in resolving imbalance issues. Future studies are needed to test the prediction performance of machine learning for imbalanced data by the classification algorithm. Fourth, this study did not investigate whether Parkinson’s disease patients took medication such as dopaminergics. Since the medication for Parkinson’s disease is related to RBD, future studies need to develop a prediction model considering medication administration.

## 5. Conclusions

This study analyzed disease data and confirmed that the prediction performance of RF was better than that of CART, artificial neural network, and regression analysis. The model for predicting Parkinson’s disease RBD developed in this study will contribute to the screening of patients who should receive an in-detail diagnosis. Based on the results in this study, further studies are required to develop a more advanced RF model that comprehensively considers the multicollinearity of input variables and issues associated with data imbalance. Furthermore, future studies should employ hybrid approaches, which have high predictive power and can interpret results.

## Figures and Tables

**Table 1:** Measurement and definition of input and outcome variables.

Factors	Measurement	Characteristics
Sociodemographic factors	Age	Continuous variable
	Sex	Male or female
	Education	Middle school graduate and below or high school graduate and above
	Handless	Left hand, right hand, or both hands
	Family dementia history	Yes or no
	Family history of Parkinson’s disease (PD)	Yes or no
	The mean age at the time of being diagnosed with Parkinson’s disease for the first time	Continuous variable
Health behaviors	Pack-years	Non-smoking, 1–20, 21–40, 41–60, or ≥61 pack-years
	Coffee consumption	Yes or no
	Mean coffee intake per day (cups/day)	No, ≤1, 2–3, or ≥4 cups
	Coffee drinking period (year)	No, ≤5, 6–9, or ≥10 years
Environmental factors	Exposure to pesticides	Never, currently not exposed but exposed previously, or currently exposed to pesticide
Disease history	Carbon monoxide poisoning	Yes or no
	Manganese poisoning	
	Traumatic brain injury	
	Diabetes	
	Hypertension	
	Hyperlipidemia	
	Atrial fibrillation	
PD related motor signs	Tremor	Yes or no
	Rigidity	
	Bradykinesia	
	Postural instability	
	Late motor complications (LMC)	
Neuropsychological characteristics	Depression	Yes or no
	Total score of K-MMSE	Continuous variable
	Total score of K-MoCA	
	Global CDR score	
	sum of boxes in CDR,	
	K-IADL	
	Total score of UPDRS	
	Motor score of UPDRS	
	H&Y staging	
	Schwab and England ADL	

K-MMSE: Korean Mini Mental State Examination; K-MoCA: Korean Montreal Cognitive Assessment; CDR: Clinical Dementia Rating; K-IADL: Korean Instrumental Activities of Daily Living; UPDRS: Untitled Parkinson’s Disease Rating; H&Y staging: Hoehn and Yahr staging; Schwab and England ADL: Schwab and England Activities of Daily Living scale.

**Table 2:** General characteristics of the subjects.

Characteristics	*n* (%)
Age, mean ± SD	68.6 ± 8.9
Age at Parkinson’s disease diagnosis, mean ± SD	68.0 ± 8.6
K-MMSE, mean ± SD	23.7 ± 4.9
K-MoCA, mean ± SD	17.6 ± 6.3
Global CDR score, mean ± SD	0.6 ± 0.5
Sum of boxes in CDR, mean ± SD	2.2 ± 2.7
K-IADL, mean ± SD	1.6 ± 3.7
Total score of UPDRS, mean ± SD	42.1 ± 21.7
Motor score of UPDRS, mean ± SD	24.1 ± 11.8
H&Y staging, mean ± SD	2.3 ± 0.7
Schwab and England ADL, mean ± SD	76.3 ± 16.4
Sex	
Male	159 (45.4)
Female	191 (54.6)
Education level	
Middle school graduate and below	216 (61.7)
High school graduate and above	134 (38.3)
Handedness	
Right hand	335 (96.3)
Left hand	8 (2.3)
Both hands	5 (1.4)
Family PD history	
No	274 (93.8)
Yes	18 (6.2)
Family dementia history	
No	257 (94.1)
Yes	16 (5.9)
Pack-year	
1–20	24 (6.9)
21–40	10 (2.9)
41–60	4 (1.1)
61 +	312 (89.1)
Coffee consumption	
No	171 (49.1)
Yes	177 (50.9)
Carbon monoxide poisoning	
No	305 (93.6)
Yes	21 (6.4)
Manganese poisoning	
No	324 (99.4)
Yes	2 (0.6)
Traumatic brain injury	
No	311 (95.4)
Yes	15 (4.6)
Diabetes	
No	277 (79.8)
Yes	70 (20.2)
Hypertension	
No	217 (62.5)
Yes	130 (37.5)
Hyperlipidemia	
No	306 (88.2)
Yes	41 (11.8)
Atrial fibrillation	
No	336 (96.8)
Yes	11 (3.2)
Tremor	
No	83 (24.6)
Yes	255 (75.4)
Rigidity	
No	25 (7.3)
Yes	319 (92.7)
Bradykinesia	
No	25 (7.3)
Yes	319 (92.7)
Postural instability	
No	162 (49.5)
Yes	165 (50.5)
Rapid eye movement sleep behavior disorder (RBD)	
No	183 (52.3)
Yes	167 (47.7)
Late motor complications	
Only on–off/wearing off	44 (13.3)
Only levodopa–induced dyskinesia	20 (6.0)
Both on-off/wearing off and levodopa-induced dyskinesia are present	38 (11.5)
Both on-off/wearing off and levodopa-induced dyskinesia are absent	229 (69.2)
Depression	
No	138 (59.0)
Yes	96 (41.0)

K-MMSE: Korean Mini Mental State Examination; K-MoCA: Korean Montreal Cognitive Assessment; CDR: Clinical Dementia Rating; K-IADL: Korean Instrumental Activities of Daily Living; UPDRS: Untitled Parkinson’s Disease Rating; H&Y staging: Hoehn and Yahr staging; Schwab and England ADL: Schwab and England Activities of Daily Living scale.

**Table 3:** The change in the out-of-bag (OOB) error rate according to the number of mtry.

Number of mtry	Error of Out-of-Bag
5	31.13%
6	30.08%
7	30.61%
8	29.44%
9	29.48%
10	29.56%
11	30.81%
12	30.83%
13	30.15%
14	30.13%
15	30.10%

The number of mtry is the number of explanatory variable candidates. The error of the out-of-bag is a method of measuring the prediction error of machine learning models utilizing bootstrap aggregating (bagging) to sub-sample data samples used for training.

**Table 4:** Accuracy of the five classifiers.

Accuracy	RF	Discriminant Analysis	CART	Neural Network	Logistic Regression
Overall accuracy	0.71	0.66	0.47	0.78	0.55
Sensitivity	0.79	0.14	0.32	0.25	0.51
Specificity	0.67	0.90	0.80	0.95	0.62

CART: classification and regression tree.
